# Comparative transcriptome profiling of a desert evergreen shrub, *Ammopiptanthus mongolicus*, in response to drought and cold stresses

**DOI:** 10.1186/1471-2164-15-671

**Published:** 2014-08-09

**Authors:** Yaqi Wu, Wei Wei, Xinyue Pang, Xuefeng Wang, Huiling Zhang, Bo Dong, Yanping Xing, Xinguo Li, Maoyan Wang

**Affiliations:** College of Life Sciences, Inner Mongolia Agricultural University, No. 306 Zhaowuda Street, Hohhot, 010018 China; Agriculture Flagship, The Commonwealth Scientific and Industrial Research Organization (CSIRO), Canberra, ACT 2601 Australia

**Keywords:** *Ammopiptanthus mongolicus*, Drought, Cold, Transcriptome profiling, Illumina sequencing

## Abstract

**Background:**

The molecular mechanisms involved in plant tolerance to either drought or cold have been extensively studied in many plant species. However, few studies have focused on their comparisons especially using non-model plants with strong tolerance to both stresses. *Ammopiptanthus mongolicus* (Maxim. ex Kom.) Cheng f. is the only evergreen broadleaf shrub grown in the central Asian desert and it has very strong cold and drought tolerance. To provide further insights into plant tolerance, the transcriptome profiles of drought- and cold-treated *A. mongolicus* seedlings were analyzed using Illumina technology and differentially expressed genes (DEGs) were compared.

**Results:**

A comprehensive transcriptome of *A. mongolicus* was sequenced using pooled mRNA extracted from drought-, cold-stressed and unstressed seedlings as well as leaves from naturally grown shrub. These sequences were assembled into 86058 unigenes, of which 51014 unigenes had an annotated function and 2440 encoded transcription factors (TFs). Transcriptome profiles were analyzed in *A. mongolicus* seedlings after drought and cold treatments at three time points (2, 8 and 24 h). Between 3917 and 6102 unigenes were identified as DEGs at a single time point in both stresses. Among these DEGs 2028 and 2026 DEGs were common across the three time points of drought and cold treatments respectively, and 971 DEGs were co-regulated by both stresses. Functional enrichment analyses identified many common or specific biological processes and gene sets in response to drought and cold stresses. The most pronounced findings are that flavonoid biosynthesis genes were enriched in the DEGs co-up-regulated by both stresses; while membrane protein genes and genes related to chloroplast were abundant in the DEGs specifically up-regulated by drought or cold, respectively. Furthermore, the DREB, ERF, NAC and WRKY TFs were predominantly co-up-regulated by both stresses.

**Conclusions:**

The present study provides the most comprehensive transcriptome resource and the first dynamic transcriptome profiles of *A. mongolicus* under drought and cold stresses. This information will deepen our understanding of plant tolerance to drought and cold. The up-regulated DEGs will be valuable for further investigations of key genes and molecular mechanisms involved in the adaptation of *A. mongolicus* to harsh environments.

**Electronic supplementary material:**

The online version of this article (doi:10.1186/1471-2164-15-671) contains supplementary material, which is available to authorized users.

## Background

Drought and low temperature are two major environmental stresses that greatly affect plant growth and crop production worldwide. This is particularly the case in arid and cold areas. To improve the tolerance of crops and other economic plants to these stresses, it is essential to know how plants respond to these stresses and which genes and biological pathways are involved in the stress tolerance. Over the past decades, thousands of genes and dozens of metabolic and signaling pathways have been identified in plants during drought and cold stresses [1–4]. Some of these genes have been confirmed to significantly improve plant tolerance to drought, cold and/or salt stresses [5, 6]. In addition, many genes have been found to be commonly or specifically regulated under drought and cold in Arabidopsis [7], rice [8] and *Brachypodium* [9] at transcriptional level. However, similar studies are very limited in other plants, and the molecular mechanisms controlling plant tolerance to both drought and cold remain largely unknown.

*Ammopiptanthus* is an endangered survivor from the Tethys in the Tertiary Period, and is the only evergreen broadleaf shrub grown in the central Asian desert. The genus *Ammopiptanthus* (Leguminosae) comprises of two species: *A. mongolicus* (Maxim. ex Kom.) Cheng f. and *A. nanus* (M. Pop.) Cheng f. Both species are diploid with 18 chromosomes and have high stress tolerance [10]. The habitats of *A. mongolicus* are marked by arid climate with an annual precipitation less than 200 mm but a mean annual evaporation up to 3000 mm. Its natural distribution areas are also characterized as sandy or stony soil with high salinity, intense ultraviolet irradiation, and seasonally extreme temperature from about –30°C in winter to more than 40°C in summer. The extreme tolerance of this species to harsh environments makes it invaluable for exploring key stress-tolerant genes and mechanisms, especially those involved in cold and drought tolerance.

The transcriptomes of *A. mongolicus* roots (treated by 20% PEG-6000 for 72 hours) and seedlings (cultured at normal condition or 4°C for 14 days) were recently sequenced by 454 pyrosequencing [11] and Illumina technology [12], respectively. These studies generated 29056 and 82795 unigenes and identified 32728 cold-regulated genes. However, transcriptome profiling of this species under drought stress and its comparison with cold stress have not been reported in the literatures.

In the present study, we firstly performed a comprehensive transcriptome sequencing of *A. mongolicus* using pooled mRNA extracted from drought-treated, cold-treated and control seedlings as well as young leaves of plants grown in desert in both summer and winter. We then applied RNA-Seq (RNA sequencing) to investigate seven cDNA libraries derived from the seedling samples exposed to drought and cold stresses for 2 h, 8 h and 24 h, and the non-stressed control seedlings respectively. Finally, we analyzed the DEGs and identified common and specific DEGs during drought and cold treatments. Our study provided the first transcriptome profile of *A. mongolicus* under drought stress and the comparison of it with the transcriptome profile under cold stress.

## Results

### Sequencing, *de novo*assembly and functional annotation of the *A. mongolicus*transcriptome

**Figure 1:**
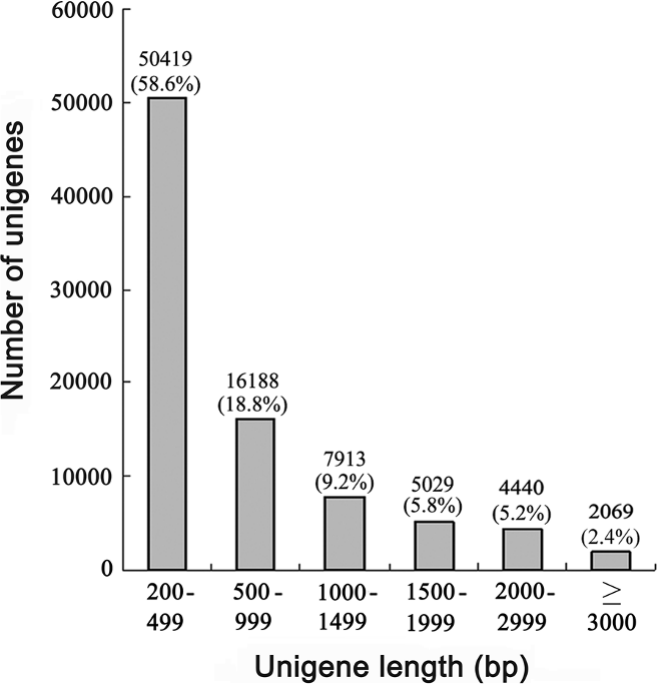
**Size distribution of the unigene sequences.** The numbers of six groups of unigenes with different lengths (200 to 499 bp, 500 to 999 bp, 1000 to 1499 bp, 1500 to 1999 bp, 2000 to 2999 bp and ≥ 3000 bp) were shown, respectively. The percentages of the unigenes in each group out of the total unigenes (86058) were also indicated.

Illumina sequencing of the pooled mRNA generated 47.29 million (M) clean paired-end reads with Q20 over 98%. *De novo* assembly of the clean reads resulted in 86058 unigenes with an average length of 756 bp and N50 length of 1279 bp (Table 1). Approximately 41.4% and 22.6% of these unigenes had a length ≥ 500 bp or ≥ 1000 bp, respectively (Figure 1).

**Table 1:** **Overview of the transcriptome sequencing and assembly**

Item	Reads	Contigs	Unigenes
Total number of raw reads	50477558	-	-
Total number of clean reads	47291846	-	-
Total clean nucleotides (bp)	4256266140	-	-
Q20 percentage (%)	98.36	-	-
Percentage (%) of assembled reads	99.84	-	-
Number of contigs or unigenes	-	190185	86058
Average length (bp)	-	325	756
N50 (bp)	-	525	1279

Of the 86058 sequences, 51014 (59.3%) unigenes had at least one significant match in the NCBI protein databases (E-value ≤ 1e-5). Distribution of the annotated unigenes in each database was shown in the Additional file 1. By searching against the PlantTFDB (http://planttfdb.cbi.edu.cn) (E-value ≤ 1e-5) in combination with annotations in the NR (non-redundant) and Swiss-Prot databases, 2440 unigenes were annotated as transcription factors (TFs), representing 4.8% of the annotated unigenes and covering 57 TF families (Table 2). Furthermore, 47486 unigenes had significant hits in the TIGR Plant Transcript Assemblies database (E-value ≤ 1e-5). Most of these hits were from *Glycine max,* one of its closest relatives*,* followed by *Medicago truncatula*, *Lotus japonicus*, *Malus x*, *Glycine soja*, and others. Surprisingly, only 919 (1.9%) and 787 (1.7%) unigenes had significant similarity to the sequences of *Arabidopsis thaliana* and *Oryza sativa*, respectively (Additional file 2).

**Table 2:** **Transcription factor (TF) gene families identified in the** ***A. mongolicus*** transcriptome*

TF family	Number of unigenes	TF family	Number of unigenes	TF family	Number of unigenes
bHLH	243	GTF	39	Nin-like	9
MYB	196	HSF	38	HB-PHD	8
AP2/EREBP	172	Dof	35	WOX	7
C2H2	156	JUMONJI	33	YABBY	7
MYB-related	155	TCP	32	BES1	6
WRKY	149	LBD	27	Whirly	6
bZIP	127	NF-YC	25	GeBP	4
C3H	82	SBP	24	SRS	4
GRAS	74	NF-YA	18	PLATZ	4
G2-like	68	ZF-HD	18	BBR/BPC	3
NAC	60	GRF	17	EIL	3
CAMTA	59	CO-like	16	S1Fa-like	3
FARI	57	NF-YB	16	VOZ	3
TALE	57	DBB	15	LIM	2
HD-ZIP	56	E2F-DP	15	NF-X1	1
ARF	54	M-type	14	STAT	1
Trihelix	52	MIKC	12	KAN	1
B3/ABI3	43	ARR-B	11		
GATA	42	TUB	11		
HB-other	40	CPP	10		

*A total of 2440 TF unigenes were identified, covering 57 TF families.

**Figure 2:**
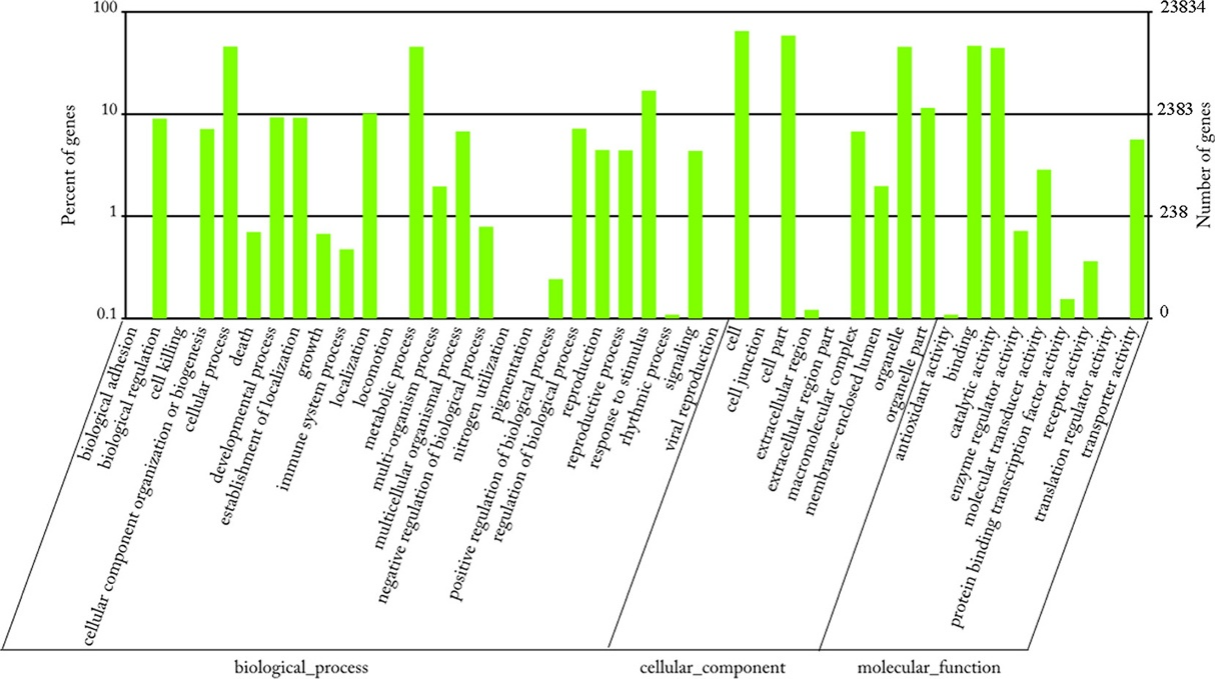
**Histogram of GO classification of the annotated unigenes.** Unigenes with best BLAST hits were aligned to GO database. In total, 23834 unigenes were assigned to at least one GO term and were grouped into three main GO categories and 44 GO terms. Left Y-axis represented the percentages of unigenes in each main category. Right Y-axis indicated the numbers of unigenes in each GO term.

Of the 86058 unigenes, 23834 unigenes were assigned to 44 GO (Gene Ontology) terms. The terms “Cell”, “Cell part”, “Organelle”, “Binding”, “Catalytic activity”, “Metabolic process”, “Cellular process” and “Response to stimulus” were mostly dominant (Figure 2). Moreover, 17100 unigenes were classified into 25 COG (Clusters of Orthologous Groups) classes. The class “General function prediction only” represented the largest group, followed by the classes “Transcription”, “Signal transduction mechanisms” and “Amino acid transport and metabolism”, *etc*. Notably, nearly 1600 of the annotated unigenes were assigned to the class of “Function unknown” (Additional file 3).

To gain insights into function interactions among the unigenes, 26999 of the 86058 unigenes were assigned to 125 KEGG (Kyoto Encyclopedia of Genes and Genomes) pathways. Of the top 10 pathways (Table 3), “Metabolic pathways” were mostly represented, followed by “Biosynthesis of secondary metabolites”, “Plant-pathogen interaction” and “Plant hormone signal transduction”, *etc*. Unigenes encoding core components in the plant hormone signal transduction pathways were present, including ABA (abscisic acid), BR (brassinosteroid) and ethylene signaling pathways. These pathways were well-documented to participate in the response and adaptation of plants to stressful environments [13, 14].

**Table 3:** **The top 10 enriched pathways in the** ***A. mongolicus*** transcriptome*

Pathway	Number of unigenes	Percentage (%)	Pathway ID
Metabolic pathways	5623	20.8	ko01100
Biosynthesis of secondary metabolites	2723	10.1	ko1110
Plant-pathogen interaction	1753	6.5	ko04626
Plant hormone signal transduction	1740	6.4	ko04075
Purine metabolism	1483	5.5	ko00230
RNA transport	1124	4.2	ko03013
Endocytosis	1004	3.7	ko04144
Splicesome	945	3.5	ko03040
Ribosome	908	3.4	ko03010
Glycerophospholipid metabolism	904	3.4	ko00564

*Percentage (%): number of the unigenes assigned to certain pathway/total 26999 unigenes assigned to 125 pathways.

### Identification and validation of differentially expressed genes (DEGs)

**Figure 3:**
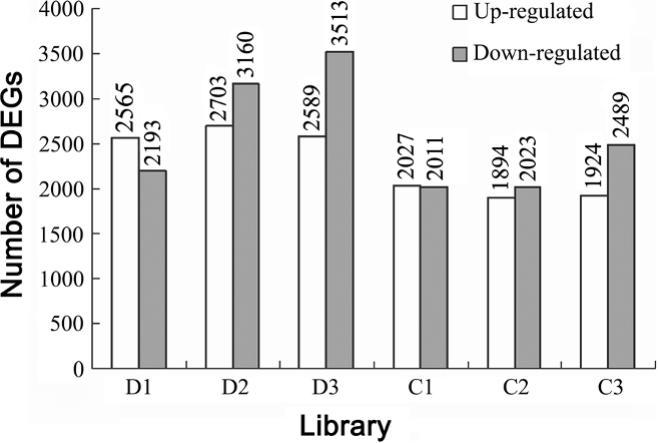
**The numbers of DEGs up- and down-regulated in each library.** The DEGs (differentially expressed genes) were identified using a threshold of FDR (false discovery rate) ≤ 0.001 and absolute value of log_2_ Ratio ≥ 1. D1, D2 and D3 represented three drought-treated libraries, corresponding to 2, 8 and 24 h after drought treatment; C1, C2 and C3 represented three cold-treated libraries, corresponding to 2, 8 and 24 h after cold treatment. The numbers of the DEGs in each library were shown in terms of up-regulation and down-regulation, respectively.

**Figure 4:**
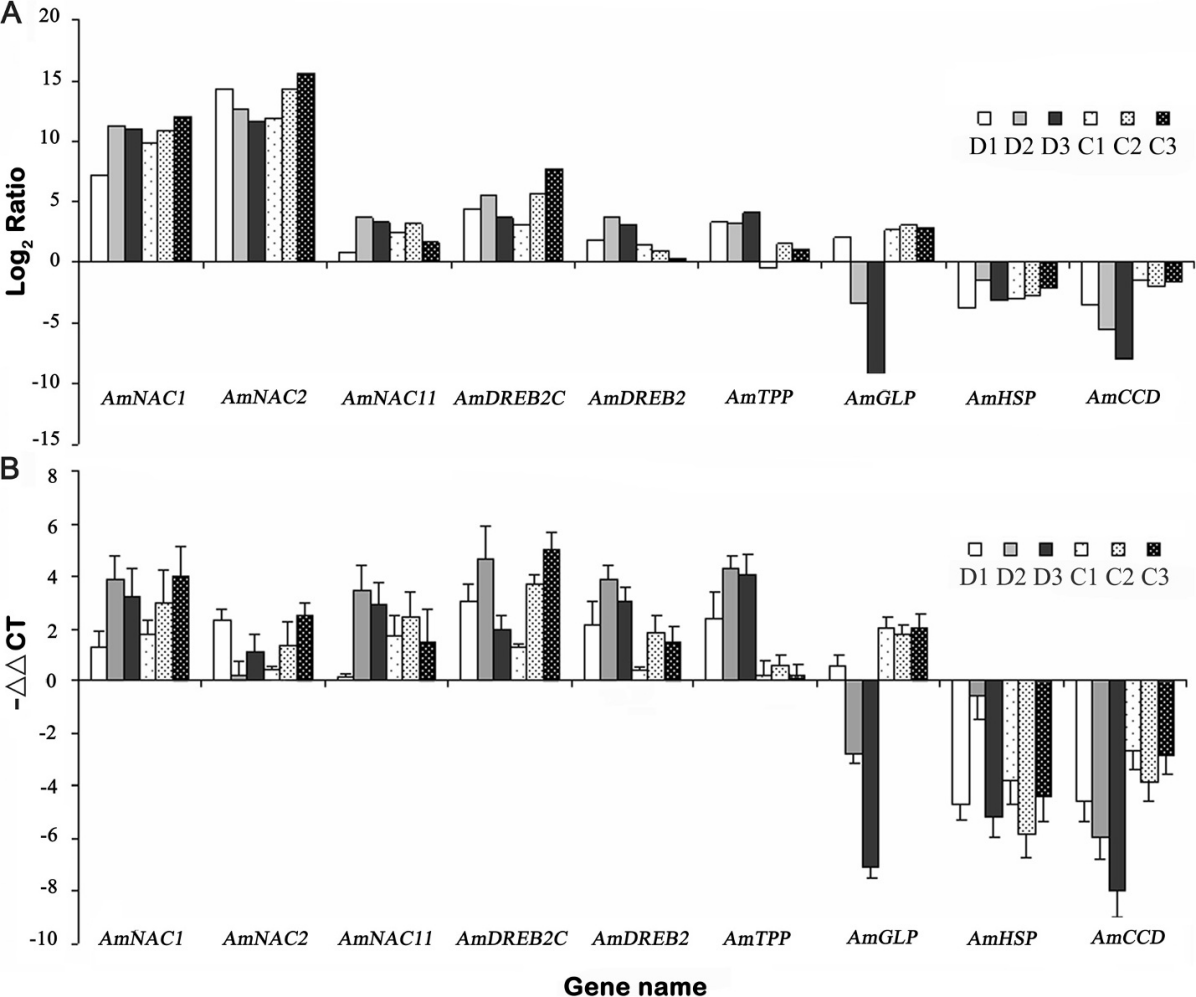
**Validation of expression patterns of nine DEGs by RT-qPCR assay.** Nine DEGs (differentially expressed genes) showed similar expression patterns between RNA-Seq data **(A)** and RT-qPCR assay **(B)**. These genes included six genes up-regulated under drought and cold stresses: *AmNAC1* (NAM, ATAF and CUC), *AmNAC2*, *AmNAC11*, *AmDREB2C* (dehydration responsive element binding 2C), *AmDREB2* and *AmTPP* (trehalose-6-phosphate phosphatase, not significantly down-regulated in C1 library); two genes down-regulated under both stresses: *AmHSP* (heat shock protein) and *AmCCD* (carotenoid cleavage dioxygenase); and one gene with different expression trends: *AmGLP* (germin-like protein). D1, D2 and D3 represented 2, 8 and 24 h after drought treatment; C1, C2 and C3 represented 2, 8 and 24 h after cold treatment. The total RNAs used for the transcriptome profile analyses using RNA-Seq were used in the RT-qPCR assay, including three biological replicates. The relative expression levels of the selected genes were calculated using the 2^–ΔΔCT^ method. Error bars represented the standard deviation of the mean expression values.

Transcriptome changes of the *A. mongolicus* seedlings under drought and cold stresses (2 h, 8 h and 24 h) and unstressed control were analyzed using Illumina technology. Between 8.29 and 8.87 M clean reads (50 bp in length) were obtained from the seven libraries. Approximately 7.18 to 7.78 M of the clean reads in each library were mapped to the 86058 unigenes assembled earlier in this study (mismatch ≤ 2 bp), accounting for 85.0% to 88.4% of the total clean reads (Table 4). In the D1, D2 and D3 libraries (2, 8 and 24 h after drought treatment), 2565, 2703 and 2589 unigenes were up-regulated and 2193, 3160 and 3513 unigenes were down-regulated, respectively. Similar results were observed in the C1, C2 and C3 libraries (2, 8 and 24 h after cold treatment), including 2027, 1894 and 1924 unigenes with up-regulation and 2011, 2023 and 2489 unigenes with down-regulation, respectively (Figure 3). Taken together, 9487 and 6524 unigenes were identified as DEGs with an absolute value of log_2_ Ratio ≥ 1 and FDR (false discovery rate) ≤ 0.001 at one or more time points during drought and cold stresses, respectively.To validate the RNA-Seq results, 11 DEGs with different expression patterns were selected for RT-qPCR (real-time quantitative PCR) analysis. These genes encode NAC (NAM, ATAF and CUC) and DREB (dehydration responsive element binding) TFs, HSP (heat shock protein), GLP (germin-like protein), TPP (trehalose-6-phosphate phosphatase) and CCD (carotenoid cleavage dioxygenase). Nine (81.8%) of these genes showed similar expression patterns to those detected by the RNA-Seq data (Figure 4), while other two genes had similar expression trends at one to two time points. Thus, the RNA-Seq results were considerably reliable for the identification of DEGs during drought and cold stresses in this study.

**Table 4:** Statistics of seven libraries and their reads mapping*

Library	CK	D1	D2	D3	C1	C2	C3
Raw reads (M)	8.91	8.34	8.66	8.92	8.59	8.38	8.37
Clean reads (M)	8.86	8.29	8.62	8.87	8.52	8.32	8.32
Mapped reads (M)	7.53	7.31	7.62	7.78	7.42	7.18	7.23
% of mapped reads	85.0	88.2	88.4	87.7	87.1	86.3	86.9
Perfectly matched reads (M)	6.25	6.12	6.37	6.53	6.22	5.90	5.98
% of perfectly matched reads	71.6	73.8	73.9	74.2	72.9	70.6	71.9
Mismatched reads (M)	1.28	1.19	1.25	1.25	1.20	1.29	1.24
% of mismatched reads	14.7	14.4	14.5	14.2	14.1	15.5	15.0
Uniquely matched reads (M)	5.60	5.27	5.54	5.75	5.46	5.27	5.31
% of uniquely matched reads	63.2	63.6	64.3	64.8	64.1	63.3	63.8
Unmapped reads (M)	1.2	0.98	1.00	1.02	1.10	1.14	1.09
% of unmapped reads	13.5	11.8	11.6	11.5	12.9	13.7	13.1
Total unigenes	60279	61029	60149	59789	61231	62693	61568
Coverage (%)	70.0	70.9	69.9	69.5	71.2	72.9	71.5

*CK: untreated control; D1, D2 and D3: 2, 8 and 24 h after drought treatment; C1, C2 and C3: 2, 8 and 24 h after cold treatment; *M*: million; Mismatch: ≤ 2 bp; Coverage (%): total unigenes in each library/86058 assembled unigenes.

### Differentially expressed genes in response to drought stress

**Figure 5:**
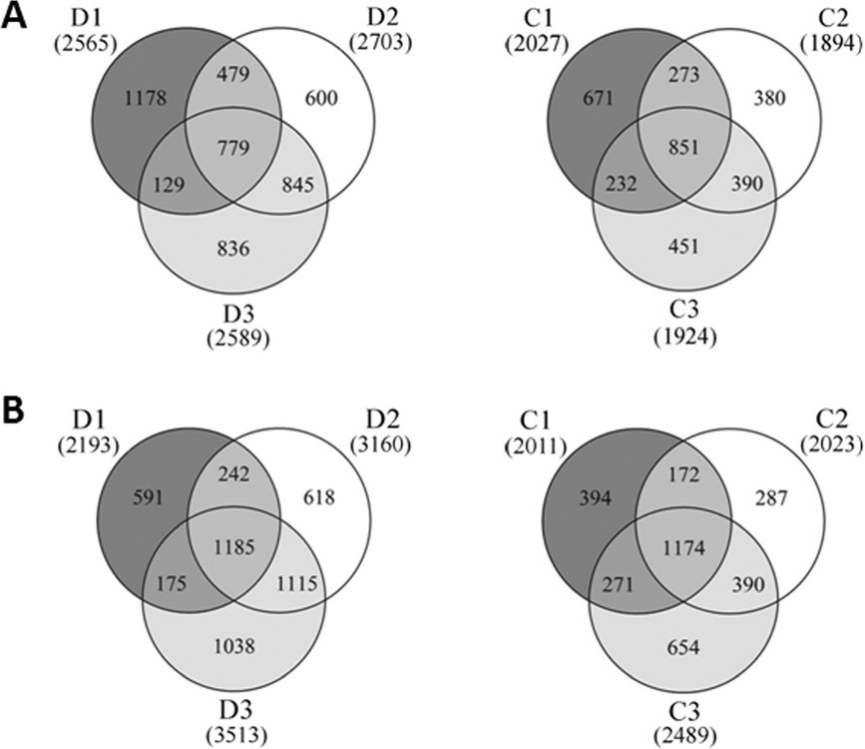
**Venn diagrams showing the numbers of common and specific DEGs at different time points of drought and cold treatments.** The up-regulated **(A)** and down-regulated **(B)** DEGs (differentially expressed genes) identified at 2, 8 and 24 h after drought (D1, D2 and D3) and cold (C1, C2 and C3) treatments were analyzed. The numbers of common and specific DEGs at different time points were shown in the overlapping and non-overlapping regions, respectively. The total numbers of up- and down-regulated DEGs at each time point were indicated in parentheses.

Of 4758 to 6102 DEGs identified at the three time points of drought treatment, 2028 DEGs (21.4% of total DEGs) were common across all three time points, including 779 DEGs with up-regulation, 1185 DEGs with down-regulation (Figure 5) and 64 DEGs with complex regulation. The common DEGs represented reproducible changes at each time point. Between 1218 and 1874 DEGs (12.8% to 19.8% of total DEGs) were specifically regulated at a single time point, including 600 to 1178 DEGs with up-regulation and 591 to 1038 DEGs with down-regulation. Besides, many DEGs were common at two of the three time points (Figure 5). The top 100 DEGs commonly regulated by drought stress were listed in the Additional file 4.

Among the common DEGs up-regulated at the three time points, genes known to respond to drought or other stresses were largely represented, such as those encoding ripening-related proteins, LEA (late embryogenesis abundant) proteins, peroxidases, transporters, enzymes in the flavonoid biosynthetic pathways, protein kinases, and ethylene receptors. Many TFs in multiple families were also up-regulated, including the AP2/EREBP (APETALA2/ethylene-responsive element binding protein), NAC, WRKY, MYB (myeloblastosis oncogene), bHLH (basic helix-loop-helix), C2H2 (Cys2/His2-type zinc finger protein 2), bZIP (basic-domain leucine-zipper protein), C3H (Cys3/His-type zinc finger protein), GRAS (GAT, RGA and SCR), TCP (TB1, CYC and PCF), COL (constans-like), Dof (DNA binding with one finger), and GRF (general regulatory factor) families, *etc*. Approximately 17.2% (134) of the common DEGs were functional unknowns. Similarly, the common DEGs down-regulated at the three time points of drought stress had a wide range of functions. For example, genes encoding aquaporin NIP, polyol transporter, gibberellin, small heat shock proteins, calcium-binding proteins, and some bHLH TFs appeared in this group. Interestingly, the unknowns in the down-regulated DEGs (397, 33.5%) were almost twice as those in the commonly up-regulated DEGs.

Based on GO enrichment analysis, a large number of the DEGs commonly up-regulated in drought were mostly enriched in two similar biological processes: “Response to stimulus” and “Response to stress”, followed by “Organic substance transport”, “Cellular aromatic compound metabolic process” and “Protein phosphorylation”. In contrast, the commonly down-regulated DEGs were significantly enriched in the biological processes “Monocarboxylic acid metabolism”, “Small molecule biosynthesis” and “Nucleobase transport”. In the cellular component and molecular function categories, the commonly up- and down-regulated DEGs were significantly enriched in similar terms, such as “Membrane” and “Oxidoreductase activity” (Additional file 5). KEGG pathway enrichment analysis revealed that genes involved in microbial metabolism in diverse environments and some secondary metabolite degradation were significantly enriched in both the up- and down-regulated DEGs. However, genes in the pathways for nitrogen and phenylalanine metabolisms were specifically enriched in the up-regulated DEGs; while genes involved in metabolic pathways and the pathways for starch and sucrose metabolism as well as carotenoid biosynthesis were only enriched in the down-regulated DEGs (Additional file 6).

As expected, many regulatory genes were specifically up-regulated at the first time point (2 h), such as genes encoding some types of protein kinases, calcium-binding proteins, TFs, as well as enzymes or components in plant hormone (especially ABA) biosyntheses or signaling pathways. After the treatment was prolonged (8 h to 24 h), many of these regulatory genes were no longer differentially expressed. Instead, a large number of functional genes were up-regulated, such as genes encoding osmoprotectant synthetases, dehydration-protective proteins, PR (pathogenesis-related) proteins, hydrolases, and detoxification enzymes.

### Differentially expressed genes in response to cold stress

Between 3917 and 4413 DEGs were identified at a single time point during cold treatment, and 2026 DEGs were common across the three time points, including 851 with up-regulation and 1174 with down-regulation (Figure 5). The top 100 DEGs commonly regulated by cold stress were listed in the Additional file 7. Similar to drought stress, many commonly up-regulated DEGs were annotated to well-known stress- or cold-induced genes. Some of these genes are identical to the drought-induced genes; while others encode different types of proteins, such as fatty acid desaturases, chitinases and proteins or enzymes located in chloroplast. The common DEGs down-regulated at three time points of cold stress also encode various functional and regulatory proteins. Notably, 16.8% (143) and 40.3% (473) of the commonly up- or down-regulated DEGs were unknowns, respectively.

A large number of the DEGs up-regulated under cold stress were significantly enriched in the biological processes “Response to stimulus”, “Response to stress”, “Defense response” and “Cellular aromatic compound metabolic process”. In the down-regulated DEGs, the mostly represented GO term of biological process was also “Response to stimulus”, followed by “Lipid biosynthetic process”, “Organic acid biosynthetic process” and “Lipid localization”. Interestingly, in the cellular component category, the up-regulated DEGs were mostly enriched in “Plastid” and “Chloroplast”; while the down-regulated DEGs were not significantly enriched in any terms of this category (Additional file 8). KEGG pathway analysis revealed that the metabolic pathways and the pathways for secondary metabolite biosynthesis or degradation were significantly enriched in both the up- and down-regulated DEGs. However, the pathways involved in phenylalanine metabolism and photosynthesis were exclusively enriched in the up-regulated DEGs. In contrast, the pathways related to fatty acid metabolism, carotenoid biosynthesis and several amino acid degradations were only enriched in the down-regulated DEGs (Additional file 9).

### Differentially expressed genes co-regulated during drought and cold stresses

Among the 9487 drought-regulated and 6524 cold-regulated DEGs with significant expression changes (absolute value of log_2_ Ratio ≥ 1 and FDR ≤ 0.001) at one or more time points, 4515 DEGs were co-regulated by both stresses, accounting for 47.6% and 69.2% of the total DEGs, respectively. Of the co-regulated DEGs, 971 DEGs had significant expression changes at all drought- and cold-treated time points, including 334 with up-regulation, 618 with down-regulation and 19 with complex regulation patterns. The results clearly showed an overlap between the DEGs in response to drought and cold. The top 100 co-regulated DEGs were listed in the Additional file 10.

Of the 971 co-regulated DEGs, 69 DEGs (33 up- and 36 down-regulated) were annotated as TFs, including the AP2/EREBP, NAC, WRKY, MYB, bHLH, C2H2, bZIP, GRAS, C3H, Dof, TCP, COL and GRF families. In addition, 46 co-regulated DEGs were annotated as protein kinases and phosphatases, and 17 co-regulated DEGs as synthetases or components in plant hormone biosynthesis or signaling. Besides, many stress-related functional genes appeared in the co-up-regulated DEGs, such as those encoding the enzymes in flavonoid biosynthetic pathways, ripening related proteins, thaumatin-like or osmotin-like proteins, transporters, cell wall-associated proteins, and some types of PR proteins.

The co-up-regulated DEGs were significantly aggregated into two GO biological processes: response to stress and secondary metabolite (mainly aromatic compounds or phenylpropanoids) biosynthesis or metabolism. However, the co-down-regulated DEGs were significantly enriched in carboxylic acid metabolic process and fatty acid or lipid biosynthetic process. In the molecular function category, the co-up- or co-down-regulated DEGs were mainly related to catalytic activity or oxidoreductase activity. Unexpectedly, both the up- and down-regulated DEGs were not significantly enriched in any cellular component terms (Table 5).

**Table 5:** Significantly enriched GO terms in the DEGs co-regulated by drought and cold*

GO term	DEGs ^a^	Unigenes ^b^	Corrected ***P***-value
**Co-up-regulated**			
**Biological process**			
Response to stress	36 (21.4%)	2565 (11.8%)	0.05017
Cellular aromatic compound metabolic process	15 (8.9%)	418 (1.9%)	0.00018
Defense response	15 (8.9%)	580 (2.7%)	0.00935
Secondary metabolic process	11 (6.5%)	232 (1.1%)	0.00040
Aromatic compound biosynthetic process	10 (6.0%)	154 (0.7%)	6.91E-05
Phenylpropanoid biosynthetic process	9 (5.4%)	147 (0.7%)	0.00044
Phenylpropanoid metabolic process	9 (5.4%)	200 (0.9%)	0.00535
**Cellular component**			
External encapsulating structure	9 (8.1%)	857 (4.1%)	1
Chloroplast	7 (6.3%)	614 (2.9%)	1
Cell wall	6 (5.4%)	329 (1.6%)	0.38977
Plant-type vacuole	3 (2.7%)	109 (0.5%)	0.97678
Chloroplast stroma	2 (1.8%)	56 (0.3%)	1
**Molecular function**			
Catalytic activity	135 (76.7%)	15838 (64.3%)	0.03081
Polysaccharide binding	3 (1.7%)	14 (0.1%)	0.01477
Pattern binding	3 (1.7%)	18 (0.1%)	0.03243
Carbon-nitrogen lyase activity	3 (1.7%)	27 (0.1%)	0.11089
Alkene binding	2 (1.1%)	3 (0.0%)	0.01818
**Co-down-regulated**			
**Biological process**			
Monocarboxylic acid metabolic process	27 (6.5%)	520 (2.4%)	0.00096
Fatty acid metabolic process	24 (5.8%)	364 (1.7%)	5.22E-05
Small molecule biosynthetic process	23 (5.6%)	324 (1.5%)	2.53E-05
Organic acid biosynthetic process	22 (5.3%)	284 (1.3%)	1.04E-05
Lipid biosynthetic process	19 (4.6%)	311 (1.4%)	0.00316
Fatty acid biosynthetic process	17 (4.1%)	138 (0.6%)	4.13E-07
Nucleobase transport	9 (2.2%)	41 (0.2%)	2.18E-05
Purine nucleoside transport	5 (1.2%)	24 (0.1%)	0.02775
**Cellular component**			
Intrinsic to membrane	27 (16.3%)	2357 (11.2%)	1
Cytoplasmic vesicle	17 (10.2%)	1240 (5.9%)	0.99779
Vesicle	17 (10.2%)	1256 (6.0%)	1
Microbody part	2 (1.2%)	33 (0.2%)	1
Peroxisomal part	2 (1.2%)	33 (0.2%)	1
**Molecular function**			
Oxidoreductase activity	44 (18.6%)	2608 (10.6%)	0.01967
Nucleobase transmembrane transporter activity	6 (2.5%)	43 (0.2%)	0.00043
Arsenate reductase activity	3 (1.3%)	4 (0.0%)	0.00044

*The significantly enriched GO terms were determined using a corrected *P*-value ≤ 0.05. If no significantly enriched GO terms were identified, the top five terms were listed. ^a^number of DEGs assigned to certain GO term; ^b^number of all reference unigenes assigned to certain GO term.

According to KEGG pathway analysis, the co-up-regulated DEGs were significantly enriched in eight pathways. Most of these pathways are completely or highly overlapped and actually belong to two pathways: microbial metabolism in diverse environments and biosyntheses of secondary metabolites. In contrast, the co-down-regulated DEGs were mainly assigned to metabolic pathways and starch/sucrose metabolic pathway. The DEGs involved in carotenoid biosynthesis and bisphenol degradation were also significantly enriched (Table 6). Furthermore, about 30% of the co-regulated DEGs were unknowns or unannotated genes. They may play unique roles in response and adaptation to drought and cold environments of *A. mongolicus*.

**Table 6:** Significantly enriched KEGG pathways in the DEGs co-regulated by drought and cold*

Pathway	DEGs ^a^	Unigenes ^b^	Corrected ***P***-value	Pathway ID
**Co-up-regulated**				
Microbial metabolism in diverse environments	29 (15.3%)	2079 (8.8%)	0.03272	ko01120
Biosynthesis of secondary metabolites	28 (14.7%)	1995 (8.4%)	0.03272	ko01110
Limonene and pinene degradation	17 (9.0%)	1009 (4.3%)	0.03274	ko00903
Aminobenzoate degradation	17 (9.0%)	1030 (4.3%)	0.03274	ko00627
Bisphenol degradation	9 (4.7%)	221 (0.9%)	0.00497	ko00363
Polycyclic aromatic hydrocarbon degradation	9 (4.7%)	248 (1.0%)	0.00594	ko00624
Chloroalkane and chloroalkene degradation	6 (3.2%)	187 (0.8%)	0.03274	ko00625
Phenylalanine metabolism	5 (2.6%)	108 (0.5%)	0.03272	ko00360
**Co-down-regulated**				
Metabolic pathways	55 (26.8%)	4071 (17.2%)	0.00961	ko01100
Starch and sucrose metabolism	13 (6.3%)	594 (2.5%)	0.04050	ko00500
Carotenoid biosynthesis	7 (3.4%)	84 (0.4%)	0.00049	ko00906
Bisphenol degradation	7 (3.4%)	221 (0.9%)	0.04684	ko00363

*The significantly enriched KEGG pathways were determined using a corrected *P*-value ≤ 0.05. ^a^number of DEGs assigned to certain KEGG pathway; ^b^number of all reference unigenes assigned to certain KEGG pathway.

### Differentially expressed genes specifically responding to drought or cold stress

In total, 4972 and 2009 DEGs were specifically regulated in at least one time point during drought or cold stress, respectively. Of these DEGs, 1057 (445 up-regulated, 566 down-regulated and 46 complicatedly regulated) or 1055 (514 up-regulated, 541 down-regulated) had significant expression changes across all three time points under drought or cold stress, respectively. The top 100 drought- or cold-specific DEGs were listed in the Additional files 11 and 12.

Among the 1057 drought-specific DEGs, 91 DEGs encode TFs, 66 encode protein kinases and phosphatases, and 24 are involved in hormone or cell signaling. Compared to the co-regulated DEGs, more TF gene families were found in the drought-specific DEGs. In the 1055 cold-specific DEGs, however, fewer genes encode regulatory proteins. Some distinct differences appeared between the drought- and cold-specific DEGs. For instance, genes encoding trehalose-6-phosphate synthase, trehalose-6-phosphate phosphatase and early responsive to dehydration protein were only found in the DEGs specifically up-regulated in drought; while genes encoding acidic endochitinase, protochlorophyllide reductase, protein chloroplast import apparatus 2 and chloroplast omega-3 fatty acid desaturase appeared only in the DEGs specifically up-regulated in cold. In addition, about 23.7% of the drought-specific DEGs and 32.3% of the cold-specific DEGs were functional unknowns.

The DEGs specifically up-regulated in drought were mostly enriched in the GO terms “Response to stress”, “Response to stimulus”, “Membrane” and “Antioxidant activity” (Additional file 13). However, the DEGs specifically up-regulated in cold were mostly enriched in the terms “Aminoglycan catabolic process”, “Plastid”, “Chloroplast” and “pattern binding” (Additional file 14). On the other hand, the DEGs specifically down-regulated in drought were more enriched in the GO terms “Small molecule biosynthetic process”, “Organic acid biosynthetic process” and “Iron ion binding” (Additional file 13); while the DEGs specifically down-regulated in cold were highly enriched in the GO terms “Lipid localization” and “Antioxidant activity” (Additional file 14). These results indicated that distinct molecular mechanisms were involved in *A. mongolicus* seedlings under drought compared to those in cold stress.

## Discussion

*A. mongolicus* is an emerging model species for studying the response and tolerance of plants to extreme cold and drought environments. By using Illumina technology, 9309 up-regulated and 23419 down-regulated DEGs were previously identified in the cold-acclimated *A. mongolicus* seedlings [12]. Using an EST approach, 120 abiotic stress-responsive genes were identified in this species, and 82 genes were confirmed to be cold- and/or drought-inducible by qRT-PCR [15]. In the present study, we performed comparative transcriptome profiling of *A. mongolicus* in response to drought and cold and identified thousands of the DEGs regulated by each stress. In particular, 971 co-regulated DEGs by both stresses and about 1050 DEGs specifically regulated by a single stress were identified, suggesting common and distinct molecular mechanisms underlying the response to drought and cold environments. These results were similar to those found in Arabidopsis [7], rice [8] and *Brachypodium* [9] despite of some new information also observed in the present study. The co-regulated DEGs could represent a subset of basal drought and cold responsive genes in *A. mongolicus* and thus may play essential roles in its adaptation to adverse environments; while the specifically regulated DEGs might underline the distinct mechanisms of this species in response to different stresses. Functional enrichment analyses of these DEGs provided clues to the molecular bases of *A. mongolicus* combating drought and cold environments.

### Flavonoids may contribute greatly to the adaptation of *A. mongolicus*to drought and cold environments

Flavonoids constitute a large group of polyphenolic secondary metabolites in plants, which have antioxidant activity and are of prime importance for plant defense against pathogens and UV stress [16]. Recent studies have showed that flavonoids accumulated to high levels in high alpine and polar plants [17] and in *A. mongolicus* [18] during cold acclimation, suggesting their important roles in cold adaptation of these plants. The biosyntheses of flavonoids require the enzymes involved in the general phenylpropanoid pathway and its flavonoid branch pathways, including phenylalanine ammonia-lyase (PAL), cinnamate-4-hydroxylase (C4H), 4-coumarate CoA ligase (4CL), chalcone synthase (CHS), chalcone isomerase (CHI), chalcone reductase (CHR), flavanone 3-hydroxylase (F3H), dihydroflavonol-4-reductase (DFR), leucoanthocyanidin dioxygenase (LDOX), anthocyanidin synthase (ANS), isoflavone synthase (IFS), 2-hydroxyisoflavanone dehydratase (HID), isoflavone reductase (IFR), isoflavone-3’-hydroxylase (IF3H), flavonol synthase (FLS), O-methyltransferase (OMT) and UDP-glycosyltransferase (UGT), *etc* [19, 20]. In the present study, genes encoding almost all of these enzymes were not only moderately or highly expressed in *A. mongolicus* seedlings in normal condition (average RPKM is 25.3), but also were significantly up-regulated by drought and cold stresses. GO terms and pathways relevant to flavonoids were highly enriched in the co-up-regulated DEGs identified in both drought and cold stresses (Tables 5 and 6). The phenylpropanoid or flavonoid biosynthetic pathways are usually regulated by MYB and/or bHLH TFs [21, 22]. Genes encoding these TFs were also included in the co-up-regulated DEGs identified in the present study. Although their exact functions need to be characterized, our results suggest that genes involved in flavonoid biosyntheses may be crucial in the adaptation of *A. mongolicus* to drought and cold environments. An increased production of reactive oxygen species is a common consequence in plants under abiotic stresses, which usually damage cellular membranes and other cellular components resulting in oxidative stress and eventually cell death [23]. It is likely that a high level of flavonoids may protect *A. mongolicus* cells from oxidative stress originated from drought and cold, and hence they contribute to stress tolerance.

### Membrane proteins may play important roles in drought adaptation of *A. mongolicus*

In plants, membrane transport and perception systems have essential roles in maintaining cellular homeostasis under adverse environmental stresses through cell-to-cell and/or organ-to-organ communication [24, 25]. An increased expression of multiple transporter and channel protein genes in response to drought has been found in some plant species [7, 24, 26]. Over-expressing some of these genes, such as aquaporin *OsPIP2-2* [27] and ABC-transporter *AtABCG36* [28], enhanced plant tolerance to drought and/or salt stress. In the present study, the GO term “Membrane” was highly enriched in the DEGs up-regulated in drought (Additional files 5 and 13), and approximately 70 membrane protein genes were found with up-regulation under drought stress. Most of these genes were annotated to encode transporters and channel proteins, such as those for ions, sugars, organic acids, water, nitrate and phosphate, suggesting their involvement in drought adaptation of *A. mongolicus*.

Receptor-like protein kinases (RLKs), another group of membrane proteins, are important signaling components that mediate plant responses to developmental and environmental stimuli [25]. Recent studies have shown that some *RLKs*, such as Arabidopsis *RPK1*, *GHR1* and *AtCRK45*, responded to drought, ABA, salt and/or cold stresses and they were implicated in ABA and BR signaling pathways [29, 30]. Genes encoding cysteine-rich receptor-like kinases and LRR-RLKs (leucine-rich receptor-like protein kinases) were highly induced in chilling-treated *Chorispora bungeana* [31]. In the present study, several RLK genes, such as those encoding LRR-RLKs, lectin-like receptor kinase, leucine-rich repeat receptor-like tyrosine-protein kinase and G-type lectin S-receptor-like serine/threonine-protein kinase, were up-regulated in drought-stressed *A. mongolicus* seedlings. Although their functions and detailed molecular mechanisms are not clear, an increased transcription of RLK genes suggests they may play important roles in drought adaptation of *A. mongolicus*.

### Chloroplast plays a central role in cold adaptation of *A. mongolicus*

Physiological studies have confirmed that chloroplast plays a central role in plant cold acclimation and freezing tolerance, which acts as sensor of plants responding to cold and light stress as well as the target of many cold acclimation processes [32]. In the present study, genes related to chloroplast were specifically enriched in the DEGs up-regulated in cold (Additional files 8, 9 and 14), covering almost all aspects of chloroplast. The largest group of these genes was involved in photosynthesis, encoding chlorophyll a-b binding proteins, photosystem II and photosystem I components, cytochrome b6/f complex subunit VIII, thioredoxins, ribulose-1, 5-bisphosphate carboxylase/oxygenase large and small subunits, as well as protochlorophyllide reductase and Mg-chelatase. This result reinforced previous findings in woody or shrub plants with strong cold tolerance, including *A. mongolicus* [15], rhododendron [33] and *Populus euphratica* [34], in which several photosynthesis-related genes were up-regulated in cold acclimation. However, it was different from those observed in herbaceous plants like Arabidopsis [35], *Brachypodium* [9], barley [36] and tomato [37] *.* In these plants with moderate cold tolerance or cold sensitivity, significant suppression of photosynthesis-related genes was observed under cold stress. An up-regulated transcription of the photosynthesis-related genes might be an important mechanism underlying the maintenance of normal or higher photosynthetic efficiency of *A. mongolicus* during extreme cold winter.

The second group of the up-regulated genes was annotated to encode early light-induced proteins (ELIPs), 20 kDa chaperonin, chaperone protein DnaJ 11, protease Do-like 8 (DEGP8) and lipoxygenase 4 (LOX 4). ELIPs might protect chloroplasts from light-induced damage or photooxidative damage [38]. Chaperonins or chaperone proteins participate in proper folding of protein substrates, protein disaggregation and degradation [39]. DEGP8 located in the thylakoid lumen [40] is probably involved in the cleavage of photodamaged proteins. LOX 4 is involved in the production of jasmonic acid and functions in biotic stress response [41]. To our knowledge, these genes (except for chaperone protein DnaJ 11) were firstly reported with up-regulation in *A.mongolicus* under cold stress.

Two other chloroplast-related genes up-regulated in cold stress encode chloroplast omega-3 desaturase and glycerol-3-phosphate acyltransferase. Increasing evidence has indicated that both genes participate in the desaturation of fatty acids in chloroplast lipids and play crucial roles in cold response and tolerance [42, 43]. An increased expression of these genes might help to maintain suitable fluidity and stability of chloroplast membranes of *A. mongolicus* in cold environment. The remaining chloroplast-related genes up-regulated under cold stress may have roles in protein import, ion transport, ATP synthesis, starch degradation, protein synthesis and processing. All these genes could provide structural and physiological mechanisms for the protection of photosynthetic apparatus, and thus for extreme tolerance of *A. mongolicus* in very cold winter.

### Transcriptional regulatory network is involved in the adaptation of *A. mongolicus*to drought and cold environments

TFs are crucial components in stress signal transduction pathways, which directly control the expression of specific sets of stress-responsive genes [44, 45]. Of the *A. mongolicus* transcriptome, at least 160 TFs in 13 TF families were identified as DEGs during drought- and/or cold treatments, such as AP2/EREBP, NAC, WRKY and bHLH families.

DREBs and ERFs (ethylene responsive factors) are two subfamilies in the AP2/EREBP superfamily [46]. It is well-known that DREBs play central roles in the regulation of cold- and drought-responsive genes and are important to the related stress tolerance in many plant species [47]. ERFs have been recently found to participate in the response of plants to abiotic stresses, and some *ERFs* can enhance drought, salt and/or freezing tolerance of their transgenic plants [48, 49]. In the present study, 9 *DERBs* and 8 *ERFs* were up-regulated in the drought- and cold-stressed *A. mongolicus* seedlings respectively, highlighting the importance of these TFs in the adaptation of this species to drought and cold environments.

The NAC family is another group of TFs involved in the tolerance of plants to abiotic stresses, especially to drought and high salinity [50, 51]. Our study identified 8 putative NAC genes up-regulated under drought and cold. Similar results were obtained in the WRKY family, a well-known family involved in biotic-stress tolerance in plants. Recent evidence showed that the WRKY family also functions in response to abiotic stresses [52]. Our findings provide supports to these results, suggesting an important role of the NAC and WRKY families in drought and cold tolerance.

The MYB, bHLH and C2H2 families are also involved in plant abiotic stress tolerance [5, 45, 53]. In the present study, different members of these families were either up- or down-regulated under drought and cold treatments. This differed obviously from the three TF families discussed earlier which were almost up-regulated during the stress treatments. In summary, sophisticated transcriptional regulation could participate in the adaptation of *A. mongolicus* to adverse environments, and several families, especially the AP2/EREBP (DREBs and ERFs), NAC and WRKY families might be important in the processes.

## Conclusions

A comprehensive transcriptome resource is generated for *A. mongolicus* and transcriptome profiles under drought and cold stresses are characterized. Comparative transcriptome analysis identified many genes and pathways commonly or specifically regulated in the two stresses, implicating molecular basis of drought and cold tolerance in *A. mongolicus*. A number of drought- and cold-regulated genes were firstly identified in this study, including some unknown genes. These newly found genes could be important to *A. mongolicus* in combating extremely cold and drought environments in desert. Our data will facilitate further molecular studies on stress tolerance of *A. mongolicus* and provide new insights into plant adaptation to harsh environments.

## Methods

### Plant stress treatment and sampling

*A. mongolicus* seeds were collected from a single shrub grown in the desert area in Dengkou County, Inner mongolia, China. These seeds were surface-sterilized with Clorox solution and soaked in water for three days at 25°C and then sown in pots filled with sand. The pots were placed in a greenhouse at 25°C under long-day condition (16 h light/8 h dark cycle). The seedlings were watered every three days with 1/5 strength of Hoagland’s solution. Three months after germination, the seedlings were treated as follows.

For *de novo* transcriptome sequencing and assembly: the seedlings were placed into a low temperature-programmable incubator (BD-PRX-1000A; -10°C to 50°C) for cold treatment at 4°C to -8°C under a 16 h dim light/8 h dark cycle in a gradual cooling way: 4°C for 6 h, 2°C for 14 h, 0°C for 4 h, -2°C for 12 h, -5°C for 8 h, and -8°C for 4 h, in total for 48 h; the cooling rate was 0.5°C per minute. This cold treatment followed by a 3-day recovery period can lead to a slight lethal damage to some leaves of the *A. mongolicus* seedlings according to our preliminary experiments (unpublished data). The seedlings were carefully removed from sand at 1 h (4°C), 3 h (4°C), 8 h (4°C 6 h + 2°C 2 h), 16 h (4°C 6 h + 2°C 10 h), 24 h (4°C 6 h + 2°C 14 h + 0°C 4 h) and 48 h (cold treatment finished) after the initiation of the cold treatment at 4°C respectively, and washed with pre-cooled water (in a refrigerator at 3°C) and collected. Drought treatment was performed as follows: the seedlings were carefully removed from sand, washed with tap water, placed on filter paper and transferred in the same incubator for dehydration at 25°C under a 16 h dim light/8 h dark cycle. The seedlings were collected at the same time points as cold stress (after 1, 3, 8, 16, 24 and 48 h of dehydration). The untreated seedlings (0 h) were used as control (CK).

For transcriptome profile analysis: the seedlings were placed into the same low temperature-programmable incubator for cold treatment in a gradual cooling way: 4°C for 4 h, 2°C for 12 h, 0°C for 2 h, -2°C for 2 h, -4°C for 2 h, and -6°C for 2 h, in total for 24 h. The drought treatment was performed using dehydration on a filter paper as described earlier. The seedlings were sampled at 2, 8 and 24 h after the initiation of the cold or drought treatment respectively. The untreated seedlings (0 h) were used as control (CK). For each time point, four seedlings were sampled. To minimize the bias of random changes from a single sample, each time point was sampled from three independent stress treatments and the total RNA was pooled equally for further experiment.

Young leaves were sampled from a single *A. mongolicus* plant grown in the desert area mentioned above in summer (late July) and winter (late December). All samples from stress treatments, controls and the young leaves were immediately frozen in liquid nitrogen and then stored at –76°C for RNA extraction.

### RNA extraction and Illumina sequencing

Total RNA was extracted using a modified Trizol method developed by our laboratory (Trizol agent plus 2% β-ME and 3 M KAc with pH 4.8). The quantity of RNAs was verified by an ultraviolet spectrometer (*OD*_260_*/OD*_280_ ratios with 1.84 to 2.04). RNAs were dissolved in DEPC-treated H_2_O. For *de novo* transcriptome sequencing and assembly, total RNAs extracted from each sample were pooled equally, and 20 μg of the pooled RNA was used for the isolation of poly (A) + mRNA. For transcriptome profile analysis, poly (A) + mRNA was purified from 10 μg total RNA from each cold- or drought-stressed seedlings and control, respectively.

The mRNAs were isolated by using oligo (dT) magnetic beads and was interrupted into short fragments by adding fragmentation buffer. Taking these short fragments as templates, the first-strand cDNA was synthesized using random hexamer-primer and reverse transcriptase. The second-strand cDNA was synthesized using buffer, dNTPs, RNase H and DNA polymerase I. After the end reparation and ligation of adaptors, the products were amplified by PCR and purified using the QiaQuick PCR extraction kit (Qiagen, Valencia, CA) to create cDNA libraries. The cDNA libraries were sequenced by Beijing Genomics Institute (BGI)-Shenzhen (Shenzhen, China) on an Illumina HiSeq™ 2000 according to the manufacturer’s protocols. For *de novo* transcriptome assembly, the 90 PE (paired-end) strategy was adopted; while for transcriptome profile analysis, the 50 SE (single-end) strategy was used.

### Transcriptome assembly

Raw reads from the library were filtered to remove the dirty raw reads if they contain adaptors, over 5% of unknown nucleotides or over 50% of low quality bases (quality value ≤ 5). Clean reads were used for *de novo* assembly with Trinity, a short read assembling program released recently [54]. Briefly, Trinity firstly combined reads with certain length of overlap to form contigs. These reads were then mapped back to contigs. Finally, Trinity connected the contigs and generated unigenes, *i.e.* the sequences that cannot be extended on either end.

### Functional annotation and classification of the unigenes

The unigenes were aligned to protein databases in the NCBI in the priority order of NR, Swiss-Prot, KEGG and COG by BLASTX algorithm [55], with a cut-off E-value ≤ 1e-5. Proteins with the highest sequence similarity were retrieved as functional annotations for a given unigene. For unigenes that did not align to any of the above databases, ESTScan software was used to predict their coding regions and corresponding amino acid sequences [56]. To identify putative TFs, all unigenes were blasted against the plantTFDB (http://planttfdb.cbi.edu.cn) with a cut-off E-value ≤ 1e-5, and the top hit for each query was applied. Unigenes with no significant hits in the plantTFDB but with annotations as TFs in the NR and Swiss-Prot databases (E-value ≤ 1e-10) were manually selected and classified into the corresponding TF families. To ascertain the sequence similarity of *A. mongolicus* to other plant species, total unigenes were searched by BLASTN against the PlantGDB-generated Unique Transcripts (http://www.plantgdb.org/prj/ESTCluster/) (similarity ≥ 80% and E-value ≤ 1e-5).

The GO annotations of the unigenes were obtained by using Blast2GO program [57]. The WEGO software was applied to perform GO functional classification for all annotated unigenes and to show the distribution of gene functions of the species at macro level [58]. After aligning the unigenes to the COG database, the COG functional annotations were acquired. The KEGG pathway annotations were obtained by aligning the unigenes to the KEGG database [59].

### Transcriptome profile analyses

The clean reads from each library were mapped to the assembled transcriptome sequences (used as reference genes) using SOAPaligner/SOAP2 [60]. Mismatches with no more than two bases were allowed in the alignment, and ambiguous reads with multi-position match were excluded. Gene expression levels were measured by the RPKM (the numbers of reads per kilobase of exon region in a given gene per million mapped reads) values [61]. If there were two or more transcripts for a gene, the longest one was used to calculate its RPKM. To identify genes responding to drought and cold, the number of reads and RPKM for each coding region in the control and each stress-treated library were determined, and the ratios of RPKM value for each gene in six stress-treated libraries to that in control library were calculated. Statistical significance of the differential expression value for each gene was determined according to Audic and Claverie [62].

Genes were selected as DEGs according to the FDR values ≤ 0.001 and at least a two-fold change (the absolute value of log_2_ Ratio ≥ 1) in expression level between two libraries. GO enrichment analysis was performed by mapping all the DEGs to GO terms in the GO database (http://www.geneontology.org/) and the number of unigenes included in each term was calculated. Hypergeometric test [63] was used to identify significantly enriched GO terms in DEGs compared with the assembled transcriptome of *A. mongolicus* using p-value ≤ 0.05. Pathway enrichment was analyzed by mapping DEGs to the KEGG database (http://www.genome.jp/kegg). The number of unigenes involved in each pathway was calculated. By comparing with the assembled transcriptome of *A. mogolicus*, the significantly enriched pathways were identified using p-value ≤ 0.05.

### Validation of DEGs by RT-qPCR

Eleven genes with different expression patterns were chosen for the validation. The template cDNAs were synthesized from 1.5 μg of total RNAs (pre-treated with DNase I) from the same samples as those for the transcriptome profile analysis using M-MLV Reverse Transcriptase (Promega, USA). The 2 × SYBR-Green I RT-PCR Master Mix (Takara, Japan) was used as labeling agent, and *α-tubulin* of *A. mongolicus* was served as an internal reference gene. The reaction mixture (20 μL) contained 10 μL 2× Master Mix, 10 μM forward and reverse primers (Additional file 15) (0.4 μL each) and 1 μL template cDNA (diluted 10 folds with deionized water). The PCR program was as follows: 95°C for 30 sec; the first step: 40 cycles of 95°C for 5 sec, 55 to 60° for 20 sec and 72°C for 20 sec; the second step: 1 cycle of 95°C for 15 sec, 55 to 60° for 1 min, and 72°C for 15 sec. The reaction was performed on a Statagene Mx3000 PCR system. Three independent biological replicates were performed for each sample. The relative expression levels of the selected unigenes were calculated using the relative 2^–ΔΔCT^ method [64]. Results represent mean standard deviations of the three experimental replicates.

### Availability of supporting data

The data set supporting the results of this article is available in the NCBI SRA (short read archive) repository under the accession number of SRS526744 (NCBI SRA, http://www.ncbi.nlm.nih.gov/sra?term= SRS526744).

## Electronic supplementary material

Additional file 1: **Distribution of the annotated unigenes in each database.** ^a^annotated or unannotated unigenes/total unigenes × 100%; ^b^annotated unigenes in each database/total annotated unigenes × 100%. (XLS 19 KB)

Additional file 2: **Species distribution of** ***A. mongolicus*** **sequences in the TIGR Plant Transcript Assemblies database.** All unigenes were searched by BLASTN against the TIGR Plant Transcript Assemblies database. In total, 47486 unigenes had significant hits (similarity ≥ 80% and E-value ≤ 1e-5), and the sequence similarities to 12 species were shown in the figure. (TIFF 3 MB)

Additional file 3: **Histogram of COG classification of the annotated unigenes.** In total, 17100 annotated unigenes were assigned to 25 COG classes. (TIFF 3 MB)

Additional file 4: **The top 100 DEGs commonly regulated by drought stress.** The unigenes with absolute values of log_2_ Ratio ≥ 1 and FDR ≤ 0.001 were identified as DEGs. (XLS 40 KB)

Additional file 5: **The significantly enriched GO terms in the DEGs commonly regulated by drought stress.** The significantly enriched GO terms were determined using a corrected P-value ≤ 0.05. If no significantly enriched GO terms were identified, the top five terms were listed. ^a^number of DEGs assigned to certain GO term; ^b^number of all reference unigenes assigned to certain GO term. (XLS 32 KB)

Additional file 6: **The significantly enriched KEGG pathways in the DEGs commonly regulated by drought stress.** The significantly enriched KEGG pathways were determined using a corrected P-value ≤ 0.05. ^a^number of DEGs assigned to certain KEGG pathway; ^b^number of all reference unigenes assigned to certain KEGG pathway. (XLS 20 KB)

Additional file 7: **The top 100 DEGs commonly regulated by cold stress.** The unigenes with absolute values of log_2_ Ratio ≥ 1 and FDR ≤ 0.001 were identified as DEGs. (XLS 38 KB)

Additional file 8: **The significantly enriched GO terms in the DEGs commonly regulated by cold stress.** The significantly enriched GO terms were determined using a corrected P-value ≤ 0.05. If no significantly enriched GO terms were identified, the top five terms were listed. ^a^number of DEGs assigned to certain GO term; ^b^number of all reference unigenes assigned to certain GO term. (XLS 37 KB)

Additional file 9: **The significantly enriched KEGG pathways in the DEGs commonly regulated by cold stress.** The significantly enriched KEGG pathways were determined using a corrected P-value ≤ 0.05. ^a^number of DEGs assigned to certain KEGG pathway; ^b^number of all reference unigenes assigned to certain KEGG pathway. (XLS 24 KB)

Additional file 10: **The top 100 DEGs co-regulated by drought and cold.** These DEGs had absolute values of log_2_ Ratio ≥ 1 and FDR ≤ 0.001 at all drought- and cold-treated time points. (XLS 48 KB)

Additional file 11: **The top 100 DEGs specifically regulated by drought stress.** These DEGs had absolute values of log_2_ Ratio ≥ 1 and FDR ≤ 0.001 at three drought-treated time points. (XLS 40 KB)

Additional file 12: **The top 100 DEGs specifically regulated by cold stress.** These DEGs had absolute values of log_2_ Ratio ≥ 1 and FDR ≤ 0.001 at three cold-treated time points. (XLS 36 KB)

Additional file 13: **The significantly enriched GO terms in the DEGs specifically regulated by drought stress.** The significantly enriched GO terms were determined using a corrected P-value ≤ 0.05. If no significantly enriched GO terms were identified, the top five terms were listed. ^a^number of DEGs assigned to certain GO term; ^b^number of all reference unigenes assigned to certain GO term. (XLS 24 KB)

Additional file 14: **The significantly enriched GO terms in the DEGs specifically regulated by cold stress.** The significantly enriched GO terms were determined using a corrected P-value ≤ 0.05. If no significantly enriched GO terms were identified, the top five terms were listed. ^a^number of DEGs assigned to certain GO term; ^b^number of all reference unigenes assigned to certain GO term. (XLS 29 KB)

Additional file 15: **Primer sequences used in the RT-qPCR assay.** The primer pairs for 9 selected unigenes were shown. (XLS 20 KB)

## Abbreviations

TF: Transcription factor
DEG: Differentially expressed gene
NR: Non-redundant
GO: Gene Ontology
COG: Clusters of Orthologous Groups
KEGG: Kyoto Encyclopedia of Genes and Genomes
ABA: Abscisic acid
BR: Brassinosteroid
FDR: False discovery rate
RT-qPCR: Real-time quantitative PCR
NAC: NAM, ATAF and CUC
DREB: Dehydration responsive element binding
HSP: Heat shock protein
GLP: Germin-like protein
TPP: Trehalose-6-phosphate phosphatase
CCD: Carotenoid cleavage dioxygenase
LEA: Late embryogenesis abundant
AP2/EREBP: APETALA2/ethylene-responsive element binding protein
MYB: Myeloblastosis oncogene
bHLH: Basic helix-loop-helix
C2H2: Cys2/His2-type zinc finger protein 2
bZIP: Basic-domain leucine-zipper protein
C3H: Cys3/His-zinc finger protein
GRAS: GAT, RGA and SCR
TCP: TB1, CYC and PCF
COL: Constans-like
Dof: DNA binding with one finger
GRF: General regulatory factor
PR: Pathogenesis-related
PAL: Phenylalanine ammonia-lyase
C4H: Cinnamate-4-hydroxylase
4CL: 4-coumarate CoA ligase
CHS: Chalcone synthase
CHI: Chalcone isomerase
CHR: Chalcone reductase
F3H: Flavanone 3-hydroxylase
DFR: Dihydroflavonol-4-reductase
LDOX: Leucoanthocyanidin dioxygenase
ANS: Anthocyanidin synthase
IFS: Isoflavone synthase
HID: 2-Hydroxyisoflavanone dehydratase
IFR: Isoflavone reductase
IF3H: Isoflavone-3’-hydroxylase
FLS: Flavonol synthase
OMT: O-methyltransferase
UGT: UDP-glycosyltransferase
RLK: Receptor-like protein kinase
LRR-RLK: Leucine-rich receptor-like protein kinase
ELIP: Early light-induced protein
DEGP8: Protease Do-like 8
LOX 4: Lipoxygenase
ERF: Ethylene responsive factor
RPKM: Reads per kilobase per million reads
SRA: Short read archive.
