# Supplementary figures and images for: Comparative transcriptome profiling of a desert evergreen shrub, Ammopiptanthus mongolicus, in response to drought and cold stresses
Source: BMC Genomics. 2014 Aug 9;15(1):671. doi: 10.1186/1471-2164-15-671 (PMC4143566; doi:10.1186/1471-2164-15-671)

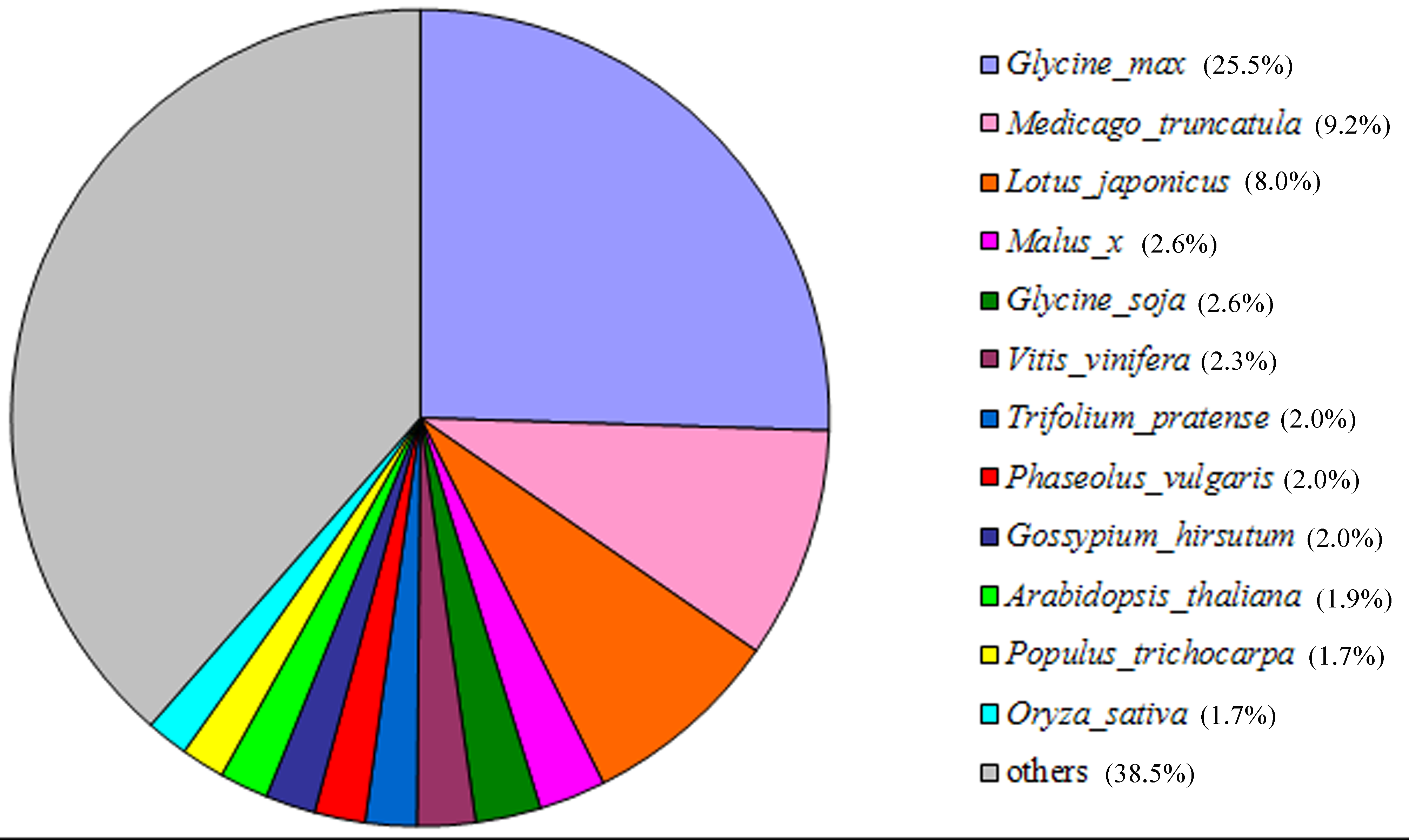

Supplement: Supplementary file 2 — Additional file 2: Species distribution of A. mongolicus sequences in the TIGR Plant Transcript Assemblies database. All unigenes were searched by BLASTN against the TIGR Plant Transcript Assemblies database. In total, 47486 unigenes had significant hits (similarity ≥ 80% and E-value ≤ 1e-5), and the sequence similarities to 12 species were shown in the figure. (TIFF 3 MB) [file 12864_2014_6369_MOESM2_ESM.tiff]

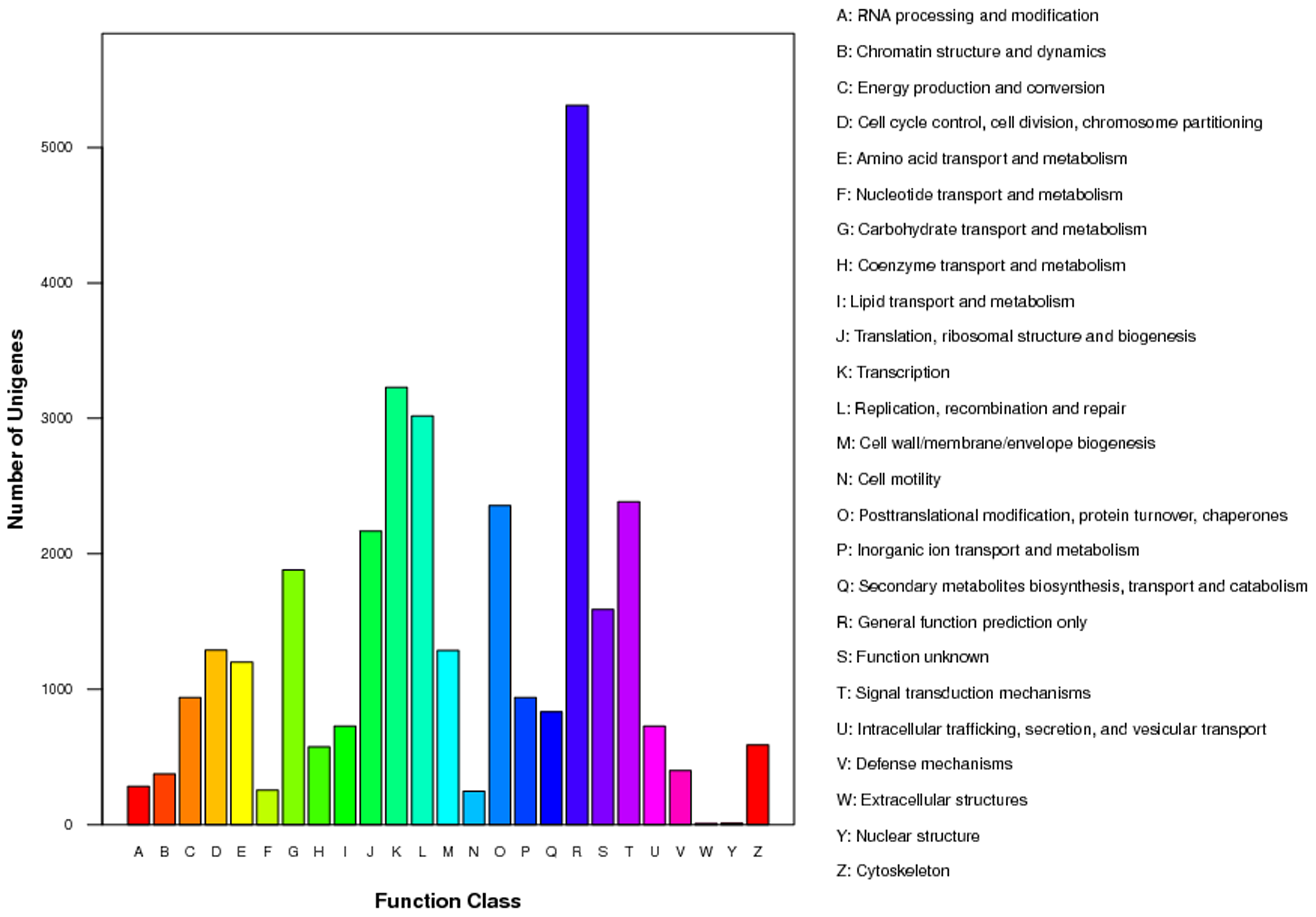

Supplement: Supplementary file 3 — Additional file 3: Histogram of COG classification of the annotated unigenes. In total, 17100 annotated unigenes were assigned to 25 COG classes. (TIFF 3 MB) [file 12864_2014_6369_MOESM3_ESM.tiff]
